# The Role of Antiplatelet Therapy in Patients With MINOCA

**DOI:** 10.3389/fcvm.2021.821297

**Published:** 2022-02-14

**Authors:** Luis Ortega-Paz, Mattia Galli, Davide Capodanno, Salvatore Brugaletta, Dominick J. Angiolillo

**Affiliations:** ^1^Division of Cardiology, University of Florida College of Medicine, Jacksonville, FL, United States; ^2^Cardiovascular Institute, Hospital Clinic, August Pi i Sunyer Biomedical Research Institute, Barcelona, Spain; ^3^Cardiovascular Medicine, Fondazione Policlinico Universitario A Gemelli Scientific Institute for Research, Hospitalization and Healthcare, Rome, Italy; ^4^Division of Cardiology, Azienda Ospedaliero Universitaria Policlinico “G. Rodolico-San Marco” University of Catania, Catania, Italy

**Keywords:** antiplatelet therapy, dual anti-platelet therapy, coronary artery disease non-obstructive, myocardial infarction, atherosclerotic plaque, coronary vasospasm, microvascular disease, spontaneous coronary artery dissection

## Abstract

Myocardial infarction with non-obstructive coronary arteries (MINOCA) is a heterogeneous group of clinical entities characterized by the common clinical evidence of myocardial infarction (MI) with non-obstructive coronary arteries on coronary angiography and without an overt cause for the MI. Platelets play a cornerstone role in the pathophysiology of MI with obstructive coronary arteries. Accordingly, antiplatelet therapy is recommended for treating patients with MI and obstructive coronary disease. However, the role of platelets in the pathophysiology of MINOCA patients is not fully defined, questioning the role of antiplatelet therapy in this setting. In this review, we will assess the role of antiplatelet therapy in MINOCA with a focus on the pathophysiology, therapeutic targets, current evidence, and future directions according to its different etiologies.

## Introduction

Invasive coronary angiography plays a crucial role in the diagnosis and management of myocardial infarction (MI). Based on coronary angiography findings, MI patients can be classified according to the presence of obstructive or non-obstructive coronary artery disease (CAD) (1). Myocardial infarction with non-obstructive coronary arteries (MINOCA) is a heterogeneous set of multiple clinical entities characterized by the common clinical evidence of MI with non-obstructive coronary arteries on coronary angiography (<50% stenosis) and without an overt cause for the MI (1). Moreover, MINOCA may represent 1–13% of all MI patients who undergo coronary angiography (2). Due to the different clinical etiologies, MINOCA has several different pathophysiological pathways, which can lead to a variety of treatment modalities. In patients with MI and obstructive coronary artery, the use of antiplatelet therapy is vastly supported by randomized controlled trials (RCTs) and forms a central part of guideline-recommendations (3). However, in patients with MINOCA, the role of antiplatelet therapy is less well-understood and may vary significantly according to the underlying etiology (1). In this review, we will assess the role of antiplatelet therapy in MINOCA with a focus on the pathophysiology, therapeutic targets, current evidence, and future directions according to its different etiologies.

## Potential Pathophysiological Pathways

**Figure 1:**
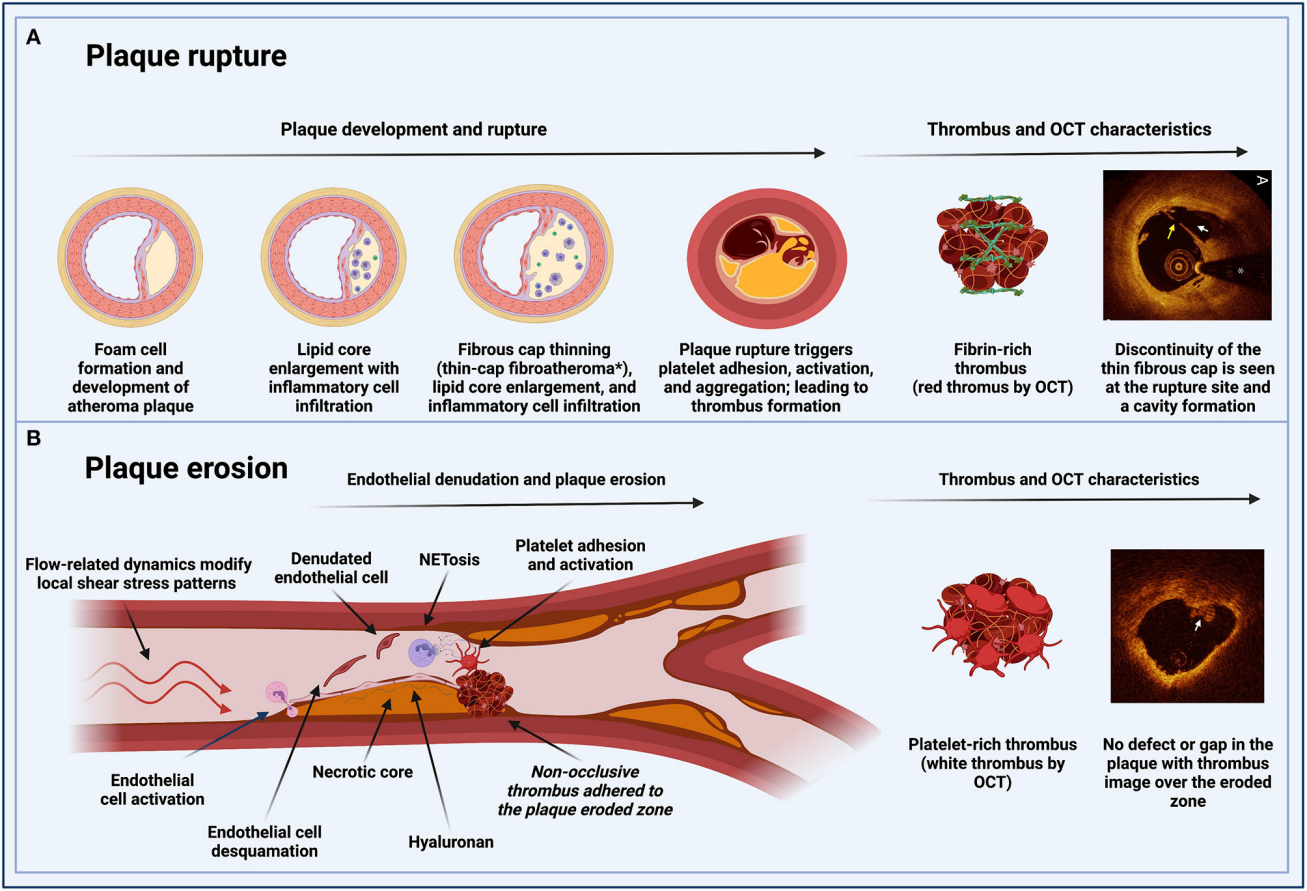
Pathophysiological pathways of MINOCA in patients with atherosclerotic disease. **(A)** Plaque rupture: inflammation is involved in the process of plaque development and rupture. Unstable plaques have a large lipid core and a thin fibrous cap with reduced collagen content. A major component of plaque destabilization appears to be increased matrix degradation, the primary regulators of which are the matrix metalloproteinase. Plaque rupture occurs where the cap is thinnest and most infiltrated by foam cells. In eccentric plaques, the weakest spot is often the cap margin or shoulder region, and only extremely thin fibrous caps are at risk of rupturing. Exposure of the thrombogenic lipid core material lead to fibrin-rich thrombus formation. **(B)** Plaque erosion: flow-related dynamics modify local shear stress patterns on the endothelial monolayer. The basement membrane (endothelial-to-mesenchymal transition) is degraded. These changes result in the desquamation of the endothelial cells from the basement membrane and their subsequent death by apoptosis. Neutrophils in the area undergo a particular type of apoptosis (NETosis) to form neutrophil extracellular traps (NETs), which produce a potent inflammatory stimulus containing tissue factor, leading to entrapment of circulating platelets and facilitating the formation of a platelet-rich thrombus. *Thin-cap fibroatheromas (TCFAs) for coronary fibroatheromas with a fibrous cap thickness of <65 μm. MINOCA, myocardial infarction with non-obstructive coronary arteries; OCT, optical coherence tomography; NET, neutrophil extracellular traps.

**Figure 2:**
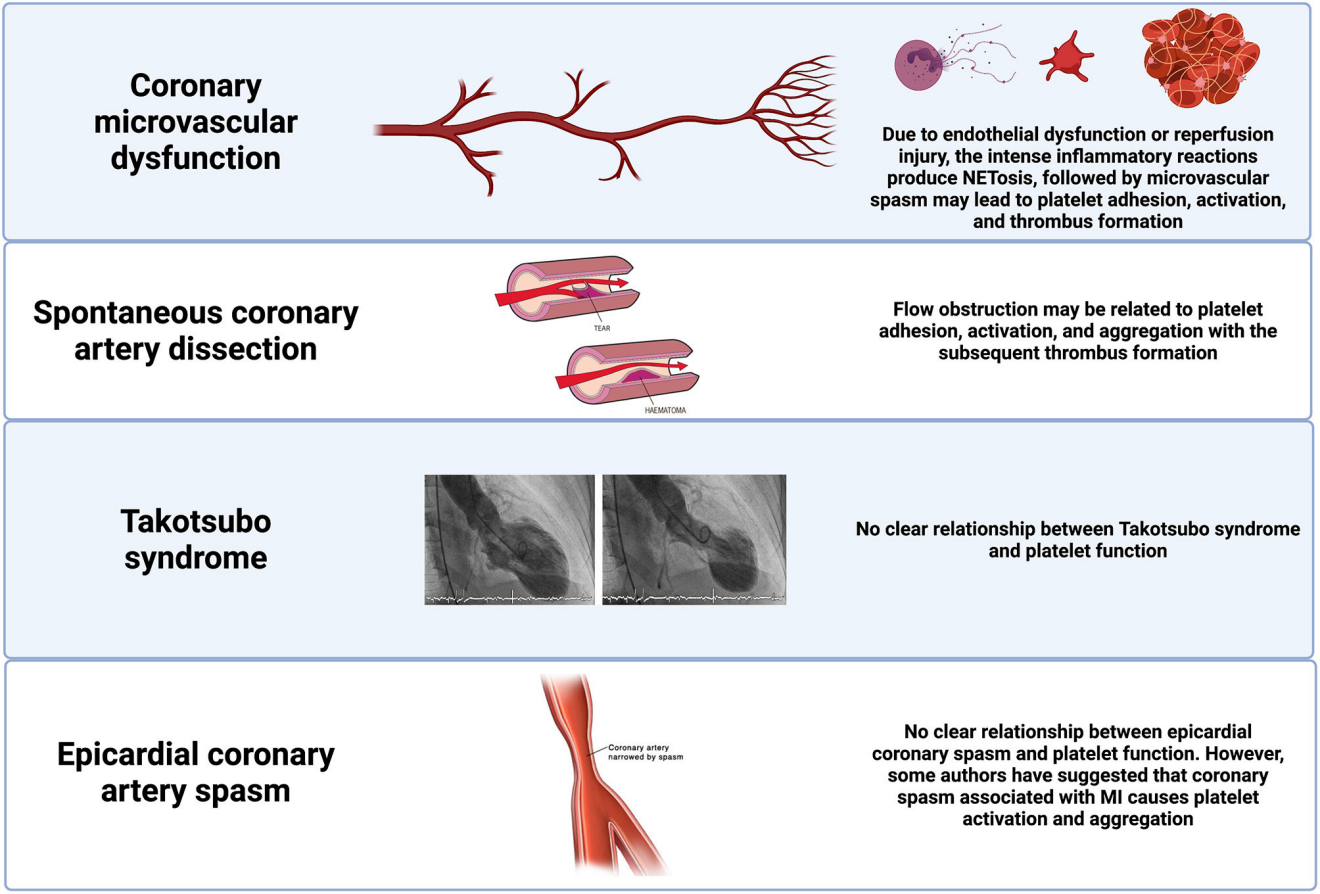
Pathophysiological pathways of MINOCA in patients without coronary atherosclerotic disease. Potential pathophysiological pathways in selected non-atherosclerotic causes of MINOCA and their relationship with platelet function. MINOCA, myocardial infarction with non-obstructive coronary arteries.

The potential pathophysiological pathways of MINOCA are directly related to the underlying etiology. Figures 1, 2 represents the different etiologies that may lead to MINOCA. MINOCA etiologies can be divided in cardiac and extra-cardiac (1). Cardiac etiologies can be classified into coronary and non-coronary causes. Furthermore, coronary causes can be classified according to the presence or not of atherosclerotic disease. However, an overlap among different mechanism is not infrequent. Among the non-coronary causes, some etiologies may be associated with myocardial disorders (i.e., myocarditis or Takotsubo syndrome). Nevertheless, others may be related to extra-cardiac conditions (i.e., stroke, respiratory failure or sepsis).

**Figure 3:**
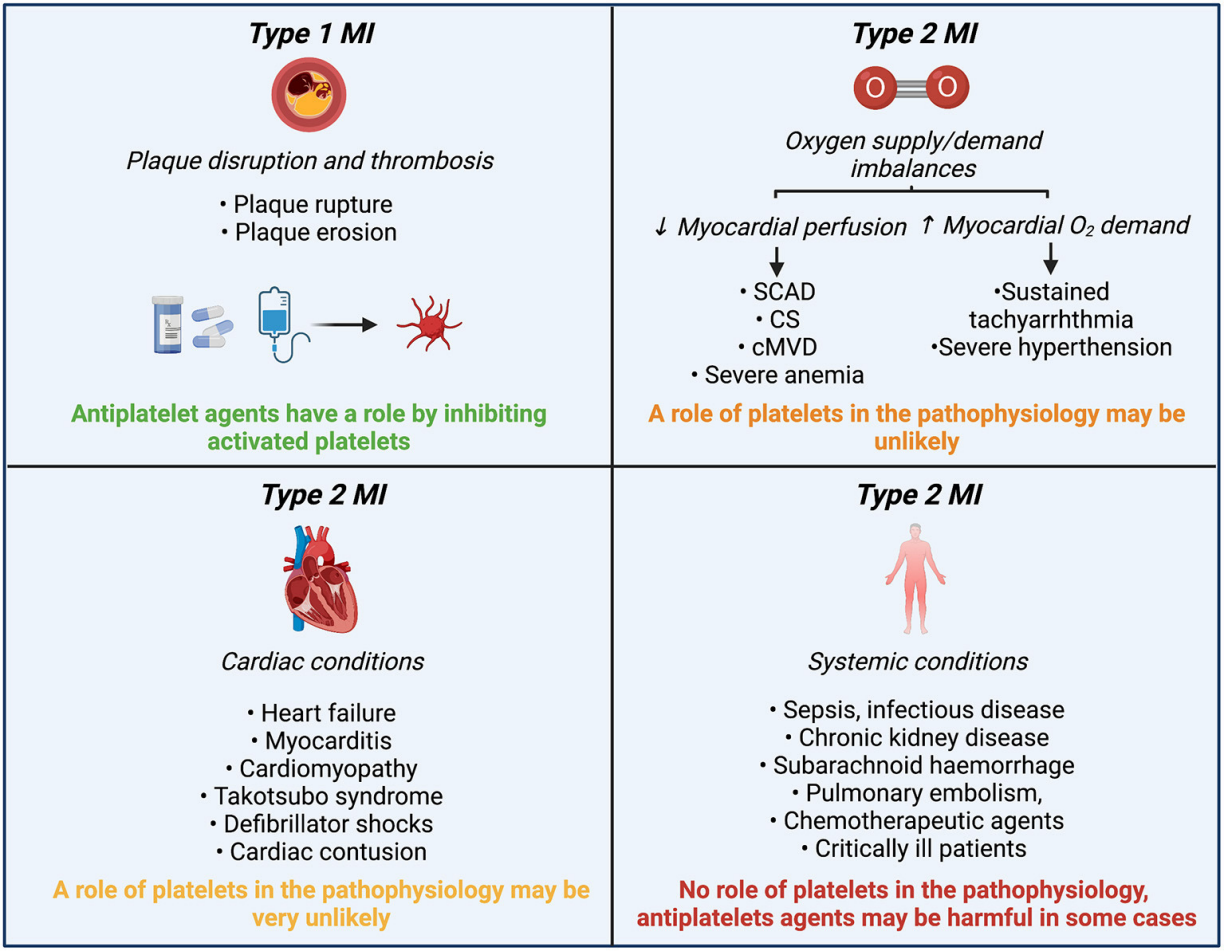
Role of platelets and antiplatelet agents in MINOCA etiologies classified according to the 4th UDMI. MINOCA, myocardial infarction with non-obstructive coronary arteries; UDMI, universal myocardial infarction definition; MI, myocardial infarction; SCAD, spontaneous coronary artery dissection; CS, epicardial coronary spasm; cMVD, coronary microvascular disease.

**Figure 4:**
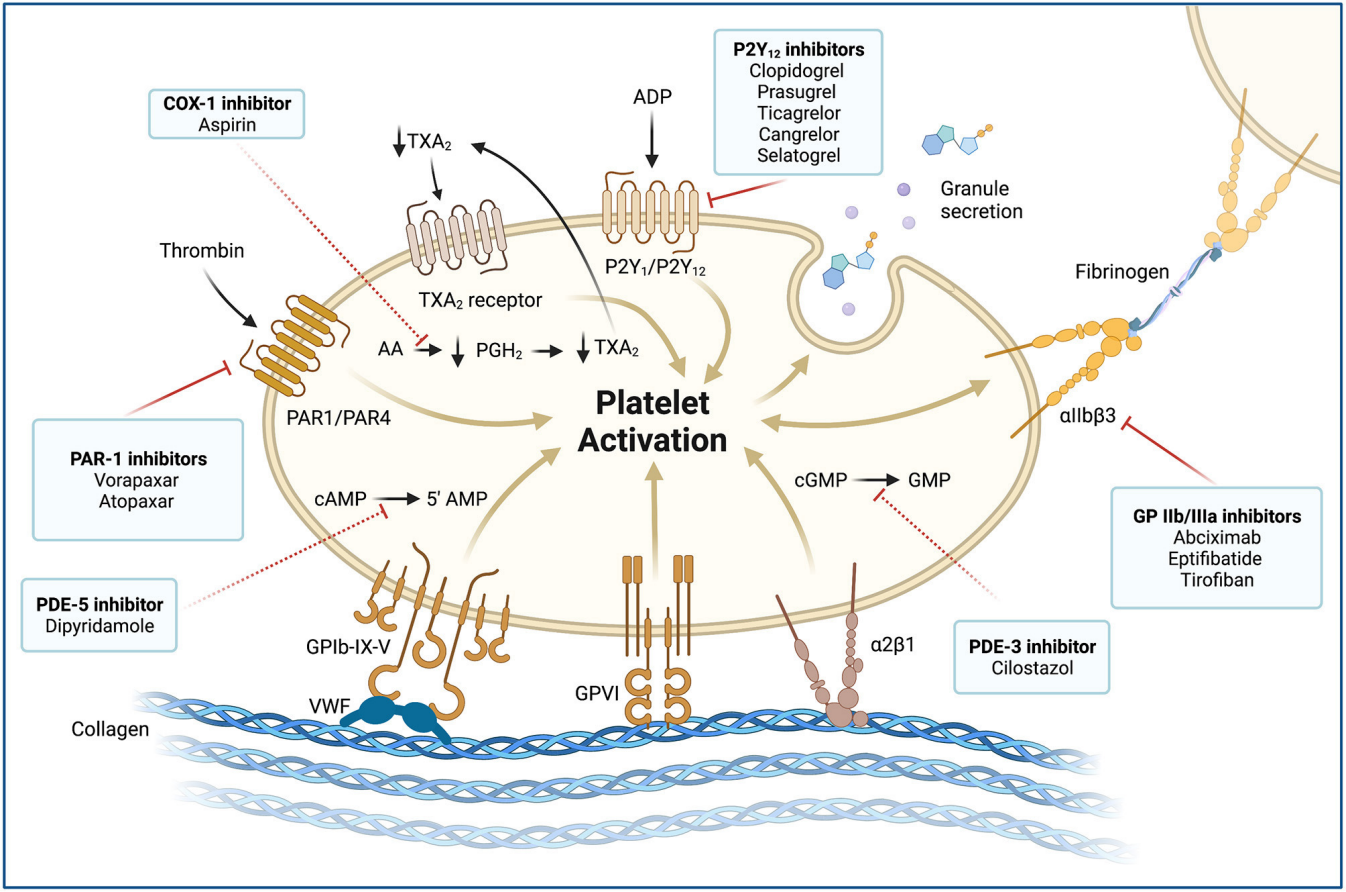
Platelet activation and molecular targets of antiplatelet agents. Antiplatelet agents and their molecular targets are shown in boxes. Blockade of membrane receptors (*continuous red lines*): Oral (clopidogrel, prasugrel, and ticagrelor) and parenteral (cangrelor and selatogrel) P2Y_12_ inhibitors block the P2Y_12_ receptor, which is a chemoreceptor for adenosine diphosphate (ADP). P2Y_12_ receptors produce a potent positive feedback loop for platelet activation. Glycoprotein (GP) αIIbβ3 inhibitors (abciximab, eptifibatide, and tirofiban) are potent parenteral antiplatelet agents. The αIIβ3 receptor is an integrin complex that is activated by platelet conformational change induced by ADP stimulus. When the αIIβ3 receptor is activated, it binds to fibrinogen and forms platelet aggregates. Proteinase-activated receptor 1 (PAR1), also called coagulation factor II (thrombin) receptor, is a G protein-coupled that plays a key role in mediating the interplay between coagulation and inflammation. PAR1 inhibitors (vorapaxar) are oral potent antiplatelet agents that inhibit the PAR1 receptor and thrombin-related platelet aggregation. Blockade of intracellular signaling pathway (*red dashed lines*): Low-dose oral aspirin exerts its effect primarily by inhibiting the cyclooxygenase (COX-1) and interfering with the cytosolic metabolism of arachidonic acid (AA) into prostaglandin H_2_ (PGH_2_) and thromboxane A_2_ (TXA_2_). TXA_2_ stimulates the thromboxane receptor, inducing platelet activation, which results in platelet-shape change, inside-out activation of integrins, and degranulation. The cytosolic reduction of TXA_2_ concentration reduces circulating TXA_2_ availability and TXA2 receptor stimulation. Phosphodiesterases (PDEs) can limit the intracellular levels of cyclic nucleotides and regulate platelet function by catalyzing the hydrolysis of cyclic adenosine 3',5'-monophosphate (cAMP) and cyclic guanosine 3',5'-monophosphate (cGMP). Dipyridamole is an oral antiplatelet agent that inhibits the PDE5 and 5'AMP production. In contrast, cilostazol is a PD3 inhibitor that blocks GMP production. MINOCA: myocardial infarction with non-obstructive coronary arteries; vWF, von Willebrand factor; GPIb-IX-V, Glycoprotein Ib-IX-V complex; GPVI, Glycoprotein VI; α2β1, Integrin α2β1.

MINOCA etiologies can also be classified according to the fourth universal MI definition (4). If underlying etiology involves the epicardial coronary arteries, these disorders may meet the type 1 MI definition. Whereas, if the etiology is due to endothelial dysfunction or oxygen supply and demand mismatch, or myocardial injury, these disorders may meet the type 2 MI definition. Although the prescription of antiplatelet therapy at discharge in MINOCA patients is frequent (69.7%), form a pathophysiological mechanism, antiplatelet agents may only have an important effect in etiologies in which platelet play a significant role (type 1 MI) (Figure 3) (5). In this review, we will focus on the etiologies that may have implications for the use of antiplatelet therapy based on potential involvement of platelets (Figure 4).

### Coronary Atherosclerotic Causes of MINOCA

#### Plaque Disruption

Plaque disruption is a common cause of MINOCA, which enclose plaque rupture, plaque erosion, and calcific nodules. Plaque disruption may cause thrombus formation, leading to an MI via distal embolization or superimposed coronary spasm. Besides, occasionally, complete transient thrombosis with spontaneous thrombolysis can be associated (1).

#### Atherosclerotic Plaque Rupture

Historically, atherosclerotic plaque rupture has been the paradigm of MI pathophysiology. Plaque rupture is the most frequent cause of atherothrombosis (55–60%). Plaque rupture is defined as a plaque with deep injury presenting defect in the fibrous cap that detached its lipid-rich atheromatous core into the coronary artery lumen, thereby exposing the thrombogenic core of the plaque (6). Coronary plaques at high risk of rupture are characterized by a lipid-rich core, thin fibrous cap with macrophage/lymphocyte infiltration, decreased smooth muscle cell content, and expansive remodeling (6). Atherosclerotic plaque rupture in large- and medium-sized arteries triggers platelet activation, leading to a fibrin-rich thrombus formation and impairment of coronary flow (Figure 1) (7). Platelet-activated thrombus proceeds in three stages, an initial phase of platelet adhesion, an extension phase that includes activation, and a perpetuation phase (7–9). In the initiation phase, platelets roll, adhere, and spread on the collagen matrix to form an activated platelet monolayer (10).

Adhesion is mediated by the interaction between the glycoprotein (GP) Ib/V/IX receptor complex on the platelet surface with von Willebrand factor (vWF) and between the GP VI and GP Ia proteins with collagen at the plaque rupture location (7). These interactions allow for activation of platelets. Under high shear conditions (i.e., small arteries, arterioles, and stenotic arteries), the interaction between vWF and GP Ib/V/IX is needed for the initial adhesion of platelets to the subendothelium (10). vWF plays a central role in platelet adhesion as it binds to both collagen and two important platelet receptors (GP Ib/V/IX and GP IIb/IIIa (αIIβ3 integrin) (10). After platelet activation, local platelet-activating factors recruit additional circulating platelets to expand and stabilize the hemostatic plug. These platelet-activating factors consist of adenosine diphosphate (ADP), thromboxane A_2_ (TXA_2_), serotonin, collagen, and thrombin (7). Thrombin is characterized for being the most potent of these factors. Importantly, thrombin is largely produced on the activated platelet surface by the prothrombinase complex and promotes the cleavage of fibrinogen into fibrin (11, 12). The release of ADP and TXA_2_ from adherent platelets contributes to the recruitment of circulating platelets and leads to distinct manifestations of platelet activation, including change in platelet shape, increased expression of proinflammatory molecules, expression of platelet procoagulant activity, and conversion of the GP IIb/IIIa receptor into an active form. The latter ultimately allows for platelet aggregation via fibrinogen bonds and vWF leading to pathological thrombosis (Figure 4) (7, 9). Of note, hemostasis in smaller vessels to stop bleeding may occur by different mechanisms, although the differences between these processes are not entirely known. Therefore, effective interventions for preventing thrombosis in the epicardial vessels may not have the same effect in smaller vessels (9).

#### Atherosclerotic Plaque Erosion

Plaque erosion is the second most common cause of atherothrombosis (30–35%). Eroded plaque is defined as a plaque with loss or dysfunction of the luminal endothelial cells leading to thrombosis. The main difference with plaque rupture is that there is no additional defect or gap in the plaque. This type of plaque is often rich in smooth muscle cells, proteoglycans and associated with constrictive remodeling (6). Moreover, plaque erosion is characterized by larger vessel lumen than in plaque rupture and the presence of a platelet-rich thrombus (13). The potential mechanism leading to thrombosis could be related to flow disturbance and inflammatory activation of the luminal endothelial cells, subsequent leukocyte recruitment, release of neutrophil extracellular traps (NETs), resulting in a cascade of thrombosis (13). Plaque erosion is possibly a weaker thrombogenic stimulus, and thrombi seem to be longer in the making than those triggered by rupture (Figure 1) (14). Ultimately, the key receptors and pathways leading to thrombosis are the same as in plaque rupture.

### Coronary Non-atherosclerotic Causes of MINOCA

#### Coronary Embolism or *in-situ* Thrombosis

Coronary thrombosis or embolism may present as MINOCA if it affects the microcirculation or if partial lysis of the epicardial coronary thrombus results in the non-obstructive angiographic pattern. Patients with hypercoagulable states may be at higher risk, but thrombosis or embolism may occur in patients without (1). Hypercoagulable disorders related to coronary thrombosis can be classified into inherited and acquired causes. The more common inherited thrombophilias are factor V Leiden and elevated factor VIII/ vWF, the prevalence of which varies according to ethnicity (15). Meanwhile, acquired hypercoagulable states include thrombotic thrombocytopenic purpura, autoimmune disorder antiphospholipid syndrome, heparin-induced thrombocytopenia, and myeloproliferative neoplasms. Although the etiology may significantly differ, the common mechanism associated with MINOCA is microvascular obstruction by microthrombus (16). Microembolization leads to platelet and inflammatory cell activation and vasospasm, which reduces coronary flow in combination with mechanical plugging of the microcirculation (17). Moreover, patients who present microvascular obstruction exhibit increased platelet activation during the acute phase and even at 1-month follow-up (18).

#### Spontaneous Coronary Artery Dissection

Spontaneous Coronary Artery Dissection (SCAD) is a relatively infrequent mechanism of MI. Nevertheless, it is a frequent cause of MI among women <50 years of age (19). Although most patients with SCAD have some degree of flow obstruction, occasionally, the arteries can appear normal or near-normal due to the distal vessel tapering. Therefore, this should be considered as a potential cause for MINOCA. In SCAD, the obstruction to coronary flow is generated by a separation of the media and adventitial vascular walls associated with intramural hematoma protrusion into the lumen. The exact pathophysiology of SCAD remains uncertain, and the primary source of the dissection (intimal or medial) is still debatable. SCAD might represent an intrinsic underlying vasculopathy that could be composite by a triggering stressor associated with a catecholamine surge, such as emotional stress, extreme physical activities, and sympathomimetic drugs. Of note, there is a significant association between SCAD and other vascular diseases (e.g., fibromuscular dysplasia) (Figure 2) (20).

#### Coronary Microvascular Disorders

The coronary microcirculation (defined as vessels < 0.5 mm diameter) is not directly visualized in the coronary angiography. However, in the absence of obstructive coronary artery disease, microcirculation accounts for ~70% of the coronary resistance (21). A standardized definition for microvascular angina has been established and enclose patients with ischemic chest discomfort, non-obstructive coronary arteries, and impaired coronary flow (21). Impaired flow can be defined according to a coronary flow reserve <2.0 in response to vasodilator stimuli, microvascular spasm diagnosed during provocative spasm testing, in the absence of epicardial coronary spasm, or impaired coronary blood flow, as measured with a corrected Thrombolysis in Myocardial Infarction (TIMI) frame count (1). In clinical practice, it is more frequent in women and patients with conventional cardiovascular risk factors (21). The initial underlying mechanism of ischemia-induced by microvascular dysfunction is endothelial dysfunction (endothelium-dependent dysfunction), which leads to vascular inflammation, platelet interaction with the adhesion cells, and activation of platelets and neutrophils. This proinflammatory environment results in microvascular spasm, impaired vasodilation, intimal thickening, and smooth cell proliferation (21). All these mechanisms lead to capillary obstruction due to capillary density and diameter reduction. Moreover, microvascular dysfunction can be a sequela of myocardial injury (endothelium-independent dysfunction) of an atherosclerotic or non-atherosclerotic etiology. Microembolization with persistent platelet activation leads to an intense inflammatory state, microvascular and tissue damage associated with neutrophil activation, and NETs production (Figure 2) (22, 23).

#### Epicardial Coronary Artery Spasm

Coronary artery spasm is defined as intense focal or diffuse vasoconstriction (i.e., >90% of the diameter) of an epicardial coronary artery deriving in impaired flow. Coronary vasospasm can occur spontaneously because of coronary vasomotor tone disorders or secondary to drugs or toxins that lead to hyperreactivity of vascular smooth muscles. Vasospastic angina is a clinical condition presenting as rest angina with a dynamic ST-segment elevation pattern on the electrocardiogram. Therefore, prolonged vasospastic episodes can lead to MINOCA. Vascular smooth muscle hyperreactivity seems to be a fundamental pathophysiological mechanism (24). However, endothelial dysfunction and vascular inflammation may also have a role in modulating this hyperreactivity (25). Some authors have proposed a link between episodes of coronary vasospasm and platelet activation (26). In particular, the rapid changes in local shear stress can potentially induce stabilized aggregation of the discoid platelets, resulting in the release of granules with vasoconstrictors. Other authors instead have suggested that coronary spasm associated with acute myocardial ischemia causes platelet activation and aggregation in the coronary circulation and may aggravate symptoms and outcomes (Figure 2) (27).

#### Takotsubo Syndrome

The underlying mechanisms of the Takotsubo syndrome (TTS) are still not completely understood. However, there is substantial evidence that sympathetic stimulation is an essential underlying pathophysiological mechanism (28). A trackable emotional or physical trigger precipitates the syndrome in most cases and has been associated with excess circulating catecholamines. Nevertheless, the exact mechanism by which catecholamines may induce myocardial stunning and regional ballooning patterns is unknown. Some of the potential mechanisms are the presence of rupture plaque on the left anterior descending coronary artery, multi-vessel epicardial spasm, microcirculatory dysfunction, and catecholamine toxicity on cardiomyocytes. Most experts disagree with the theory of ruptured plaque because intravascular imaging studies have not identified ruptured plaques in the ample majority of TTS patients (Figure 2) (29). While other studies have suggested that catecholamines and endothelin may have a role by inducing potent vasoconstriction primarily in the coronary microvasculature by stimulating the endothelin and α_1_-receptors. In clinical practice, impaired microcirculation has been found in TTS patients assessed by means of the index of microcirculatory resistance (IMR) (30). One of the most accepted mechanisms is the direct effect of catecholamines on cardiomyocytes (31). Endomyocardial biopsies can occasionally report contraction band necrosis, which is typically found in cases of extreme catecholamine production. Ultimately, it is unclear if there is an association between TTS and platelet dysfunction.

## Current Evidence

### Clinical Guidelines

Over the past decade, MINOCA has gained attention as an important clinical entity with several clinical unmet needs. In 2017, the European Society of Cardiology (ESC) cardiovascular pharmacotherapy working group published a consensus document to define MINOCA, describing its clinical features and mechanisms and stimulating research into its underlying mechanisms and treatment (16). Moreover, the 2017 ST-segment elevation Myocardial infarction (STEMI) and 2020 Non-ST-segment elevation Myocardial infarction (NSTEMI) ESC guidelines have dedicated a specific section for MINOCA (32, 33). Furthermore, the American Heart Association (AHA) in 2019 published a scientific statement document of MINOCA (1). Ultimately, Bertil et al., have provided an updated review on MINOCA, focusing on the application of different images modalities (34). Table 1 shows a summary of the key recommendations for MINOCA and a selection of its etiologies.

**Table 1:** Current clinical guidelines recommendations.

	Recommendation^*^
**MINOCA**	
Diagnostic criteria	Diagnostic is made immediately after coronary angiography in a patient presenting with features consistent with an MI and fulfilling the following criteria (32, 33): •MI defined according to the 4th Universal MI definition (4) ° Measurement of a rise or fall in cardiac troponin with at least one value above the 99th percentile upper reference limit and ° Corroborative clinical evidence of infarction as shown by at least one of the following: ▪ Symptoms of myocardial ischemia (i.e., chest pain) ▪ New ischemic electrocardiographic changes (i.e., ST-segment elevation) ▪ Development of pathological Q waves ▪ Imaging evidence of new loss of viable myocardium or new regional wall motion abnormality in a pattern consistent with an ischemic cause ▪ Identification of a coronary thrombus by angiography or autopsy.
	Defined as the absence of obstructive disease on angiography (i.e., no coronary artery stenosis ≥50%) in any major epicardial vessel ° Normal coronary arteries (no angiographic stenosis) ° Mild luminal irregularities (angiographic stenosis <30% stenosis) ° Moderate coronary atherosclerotic lesions (stenosis >30% but <50%) •No clinically overt specific cause for the acute presentation ° Alternate diagnoses include, but are not limited to, non-ischemic causes.
Diagnosis workflow (32, 33)	•In all patients with an initial working diagnosis of MINOCA, it is recommended to follow a diagnostic algorithm to differentiate true MINOCA from alternative diagnoses. (^†^ESC guidelines IC).
Diagnostic imaging (32, 33, 52)	•It is recommended to perform CMR in all MINOCA patients without an obvious underlying cause (^†^ESC guidelines IB). •In patients with acute chest pain and myocardial injury who have non-obstructive coronary arteries on anatomic testing, CMR with gadolinium contrast is effective to distinguish myopericarditis from other causes, including MINOCA (^‡^ ACC/AHA guidelines 1B-NR).
Treatment (32, 33)	•It is recommended to manage patients with an initial diagnosis of MINOCA and a final established underlying cause according to the disease-specific guidelines (ESC guidelines IC). •Patients with a final diagnosis of MINOCA of unknown cause may be treated according to secondary prevention guidelines for atherosclerotic disease (^†^ESC guidelines IIbC).
**Plaque disruption**	
Diagnosis (32, 33)	•IVUS or OCT are diagnostic tools for evaluating erosions and plaque ruptures. Due to higher resolution, OCT may be a better option for plaque erosion is suspected.
Treatment (1)	•Medical treatment: ° Aspirin ° High-intensity statin °β-blockers (in the presence of left ventricular dysfunction, and possibly with preserved EF) ° ACE inhibitors/ARBs (in the presence of left ventricular dysfunction, and possibly with preserved EF) ° Consider clopidogrel/ticagrelor •Invasive treatment: ° Stenting is a matter of debate with a not clear consensus.
**SCAD**	
Diagnosis (1)	•Extensive review of coronary angiography. •Intracoronary imaging should be considered to diagnose SCAD if suspected.
Treatment (1, 32, 33)	Optimal management is unclear since no RCTs have compared medical therapy to revascularization strategies. •According to available data, with the exception of very high-risk profile patients, a conservative approach should be the preferred strategy. •The decision to treat either with a conservative medical approach or to perform PCI or CABG surgery must be individualized and based on both clinical (persistent or recurrent angina or ischemia) and angiographic factors (LM or proximal LAD/LCx/RCA, or multivessel SCAD). •Medical treatment may include: ° Aspirin °β-blocker ° Consider clopidogrel
Follow-up (1, 33)	•Imaging: ° Among SCAD patients treated medically and having persistent or recurrent symptoms, even in the absence of recurrent MI or ischemia, CCTA might be considered for follow-up. •Lifestyles: ° Some experts recommend that patients avoid strenuous exercise and future pregnancies.
**Coronary embolism or** ***in-situ*** **thrombosis**	
Diagnosis (32, 33)	•Extensive review of coronary angiography •Intravascular imaging (HD-IVUS or OCT). OCT provides better visualization of intracoronary thrombus.
Treatment (1)	•Medical treatment: ° Antiplatelet or anticoagulant therapy ° Other targeted therapies for hypercoagulable condition
Follow-up	•Thrombophilia screen
**Coronary microcirculation dysfunction**	
Diagnosis (32, 33)	•Extensive review of coronary angiography. •Guidewire-based CFR and/or microcirculatory resistance measurements should be considered in patients with persistent symptoms, but coronary arteries that are either angiographically normal or have moderate stenosis with preserved iwFR/FFR (ESC guidelines IIaB). •Intracoronary acetylcholine with ECG monitoring may be considered during angiography if coronary arteries are either angiographically normal or have moderate stenosis with pre-served iwFR/FFR, to assess microvascular vasospasm (ESC guidelines IIbB). •Transthoracic Doppler of the LAD, CMR, and PET may be considered for the non-invasive assessment of CFR (^†^ESC guidelines IIbB).
Treatment (1)	•Medical therapy: ° Conventional antianginal therapies (e.g., calcium channel blocker, β-blocker). ° Unconventional antianginal therapies (e.g., L-arginine, ranolazine, dipyridamole, aminophylline, imipramine, α-blockers).
**Epicardial coronary spasm**	
Diagnosis (32, 33)	•An ECG is recommended during angina if possible (^†^ESC guidelines IC). •Ambulatory ST-segment monitoring should be considered to identify ST-segment deviation without increased heart rate (^†^ESC guidelines IIaC). •Invasive angiography or CCTA is recommended in patients with characteristic episodic resting angina and ST-segment changes, which resolve with nitrates and/or calcium antagonists, to determine the extent of underlying coronary disease (^†^ESC guidelines IC). •Blood toxicology testing (e.g., cocaine, methamphetamines) •Review of medication and non-prescription drug use (e.g., migraine medications). •An intracoronary provocation test should be considered to identify coronary spasms in patients with normal findings or non-obstructive lesions on coronary arteriography and a clinical picture of coronary spasm to diagnose the site and mode of spasm (^†^ESC guidelines IIaB).
Treatment (1)	•Medical treatment ° Calcium channel blockers ° Other antispastic agents (nitrates, nicorandil, cilostazol) ° Consider statin.
**Takotsubo syndrome**	
Diagnosis (28, 53)	•Left ventricular angiogram •Contrast CMR
Treatment (28, 53)	There are no RCTs to support a specific treatment and, therefore, all recommendations so far are based on expert opinions •Medical treatment: ° ACE inhibitor ° Medical (e.g., levosimendan) or device therapies (Impella or VA-ECMO) for heart failure/left ventricular dysfunction ° Consider β-blockers.

* *The selected international guidelines and consensus documents are: Contemporary Diagnosis and Management of Patients With Myocardial Infarction in the Absence of Obstructive Coronary Artery Disease: A Scientific Statement From the American Heart Association (1). ESC working group position paper on myocardial infarction with non-obstructive coronary arteries (16). 2017 ESC Guidelines for the management of acute myocardial infarction in patients presenting with ST-segment elevation (32). 2020 ESC Guidelines for the management of acute coronary syndromes in patients presenting without persistent ST-segment elevation (33). 2021 AHA/ACC Guideline for the Evaluation and Diagnosis of Chest Pain (52). International Expert Consensus Document on Takotsubo Syndrome (Part II): Diagnostic Workup, Outcome, and Management (53). 2019 ESC Guidelines for the diagnosis and management of chronic coronary syndromes (54)*.

† *European society of cardiology guidelines level of evidence and grade of recommendation*.

‡ *American Heart Association and American College of Cardiology guidelines level of evidence and grade of recommendation*.

*MI, myocardial infarction; MINOCA, myocardial infarction with non-obstructive coronary arteries; CMR, cardiac magnetic resonance; AHA, American Heart Association; ACC, American College of Cardiology; IVUS, intravascular ultrasound; OCT, optical coherence tomography; EF, ejection fraction; ACE, angiotensin-converting enzyme; ARBs, Angiotensin II receptor blockers; SCAD, spontaneous coronary artery dissection; RCT, randomized controlled trial; PCI, percutaneous cardiac intervention; CABG, coronary artery bypass graft; LM, left main artery; LAD, left anterior descending artery; LCx, left circumflex; RCA, right coronary artery; CCTA, coronary computed tomography angiography; HD-IVUS, high definition IVUS; CFR, coronary flow reserve; iwFR, instantaneous wave-free ratio; FFR, fractional flow reserve; ECG, electrocardiogram; PET, positron Emission Tomography; ESC, European society of cardiology; VA-ECMO, venoarterial extracorporeal membrane oxygenation*.

### Myocardial Infarction With Non-obstructive Coronary Arteries

Most of the reported studies have described the association between the clinical outcomes and medical therapy in overall cohorts of MINOCA patients without classifying it into the different etiologies (Table 2). One of the largest cohorts of patients was derived from the SWEDEHEART national registry (5). Between July 2003 and June 2013, the study included 9,466 consecutive patients with MINOCA. Of these, 66.4% were treated with dual antiplatelet therapy (DAPT), the use of which did not impact the composite endpoint of all-cause mortality, hospitalization for myocardial infarction, ischemic stroke, and heart failure. Furthermore, DAPT was not associated with an increase in bleeding events. Although this report has several strengths, there are also important limitations. First, MINOCA etiologies were not reported. Therefore, the specific effect of DAPT on different etiologies cannot be assessed. Second, the type of P2Y_12_ inhibitor and duration of DAPT was not reported. Ultimately, ascertainment of the primary endpoint measures were derived from the International Classification of Diseases codes.

**Table 2:** Selection of studies assessing the role of antiplatelet therapy in MINOCA.

	**Methodology**	**Results**	**Comments**
**MINOCA**			
Medical Therapy for Secondary Prevention and Long-Term Outcome in Patients With Myocardial Infarction With Non-obstructive Coronary Artery Disease (5)	- Observational study of the SWEDEHEART registry between July 2003 and June 2013 and followed until December 2013 - Primary endpoint was cardiac death. MI, stroke, heart failure, and major bleeding were also reported	−9,466 consecutive patients with MINOCA, 66.4% were treated with DAPT - DAPT have a null effect [HR 0.90, 95%CI (0.74–1.08)] on 1-year MACE - DAPT was not associated with an increase in bleeding events [HR 1.33, 95%CI (0.73–2.42)]	- Large cohort of patients evaluating medical treatment and outcomes in MINOCA. - Type of MINOCA was not reported. - Type of P2Y_12_ inhibitor and the exact duration of DAPT were not reported.
Antiplatelet therapy in patients with myocardial infarction without obstructive coronary artery disease (35)	- *Post hoc* analysis of the OASIS 7 trial - ACS patients were randomized to receive either double-dose (600 mg, day 1; 150 mg, days 2–7; then 75 mg/day) or standard-dose (300 mg, day 1; then 75 mg/day) clopidogrel. - Primary outcome was CV death, MI, or stroke at 30 days.	−23,783 patients with MI and 1,599 (6.7%) with MINOCA were included. - In MINOCA, patients allocated to clopidogrel standard-dose group had MACE risk of HR 3.57, [95%CI (1.31–9.76)]. Whereas, in those with obstructive CAD, MACE risk was HR 0.91 [95%CI (0.80–1.03); *p*-value _interaction_ = 0.011).	- An intensified dosing strategy of clopidogrel appears to offer no additional benefit with a signal of possible harm when compared to standard dosing. - Type of MINOCA was not reported. - Clopidogrel was the unique P2Y_12_ inhibitor used.
Dual antiplatelet therapy in myocardial infarction with non-obstructive coronary artery disease—insights from a nationwide registry (40)	- Multicenter Portuguese registry enrolling patients who suffered their first MI between 2010 and 2017 and underwent coronary angiography revealing the absence of stenosis ≥50%. - Determine the predictors of DAPT prescription.	−709 were categorized as MINOCA, and 390 (55.0%) were discharged on DAPT. -Males, active, previous PCI, STEMI, and sinus rhythm at admission were independent predictors of DAPT use.	- Type of MINOCA was not reported. - Type of P2Y_12_ inhibitor and the exact duration of DAPT were not reported.
Clinical and Therapeutic Profile of Patients Presenting With Acute Coronary Syndromes Who Do Not Have Significant Coronary Artery Disease (36)	- *Post hoc* analysis of the PURSUIT trial - Patients with NSTEMI who underwent coronary angiography were classified according to the presence or not of significant (≥50%) stenosis. - Primary outcome was death or non-fatal myocardial infarction at 30 days.	The frequency of death or non-fatal myocardial infarction at 30 days was reduced with eptifibatide treatment in patients with significant CAD (18.3 vs. 15.6% for placebo, *p* = 0.006) but not in those with mild CAD (6.6 vs. 5.4%, *p* = 0.62) and with no CAD (3.0 vs. 1. 2%, *p* = 0.280).	- Type of MINOCA was not reported. - Currently, with routine and potent oral P2Y_12_ receptor inhibitors, there is no compelling evidence for an additional benefit of routine upstream use of GP IIb/IIIa inhibitors in NSTEMI patients scheduled for coronary angiography.
Secondary Prevention Medical Therapy and Outcomes in Patients With Myocardial Infarction With Non-Obstructive Coronary Artery Disease (41)	- Patients with MI undergoing early coronary angiography between 2016 and 2018 were extracted from a clinical database of the Bologna University Hospital. - Primary endpoints were all-cause mortality, re-hospitalization for MI, and MACE (composite of all-cause mortality, hospitalization for MI, and ischemic stroke).	- Out of 1,141 MI who underwent coronary angiography, 134 were initially diagnosed as MINOCA, and 42.1% were treated with DAPT. - Treatment with DAPT was not associated with a reduction in all-cause mortality [HR 0.48, 95%CI (0.14–1.64)] or MACE [HR 0.42, 95%CI (0.14–1.24)]	- Type of MINOCA was not reported. - Type of P2Y_12_ inhibitor and the exact duration of DAPT were not reported.
Comparison of Patients With Non-obstructive Coronary Artery Disease With vs. Without Myocardial Infarction [from the VA Clinical Assessment Reporting and Tracking (CART) Program] (37)	- Patients who underwent coronary angiography in the Veteran Affairs system between 2008 and 2017 were classified as those with MINOCA and non-obstructive CAD without MI. - Primary endpoint MACE (all-cause death, MI, and revascularization) at 12-month.	- Out of 3,924 MI who underwent coronary angiography, 1,986 were diagnosed as MINOCA, and 20% were treated with DAPT. - Treatment with DAPT was not associated with a reduction in MACE [HR 1.02, 95%CI (0.58–1.80)].	- Type of MINOCA was not reported. - Type of P2Y_12_ inhibitor and the exact duration of DAPT were not reported. - Analysis from a selected population.
Pharmacological therapy for the prevention of cardiovascular events in patients with myocardial infarction with non-obstructed coronary arteries (MINOCA): Insights from a multicentre national registry (38)	- Multicenter Italian retrospective registry of patients discharged with MINOCA diagnosis from 2012 to 2018. - Primary endpoint was a composite of all-cause death or MI heart failure hospitalization or stroke; at 90 days.	- A total of 621 patients were included, and 58.8% were treated with DAPT. - DAPT was not associated with a reduction in the primary endpoint [HR 1.04, 95%CI (0.68–1.59)].	- Type of MINOCA was not reported. - Type of P2Y_12_ inhibitor and the exact duration of DAPT were not reported. - Analysis from a selected population.
Effect of Secondary Prevention Medication on the Prognosis in Patients With Myocardial Infarction With Non-obstructive Coronary Artery Disease (39)	- Single-center retrospective registry of patients diagnosed with MINOCA between 2014 to 2018. - Primary endpoint was MACE (composite of CV death, non-fatal MI, stroke, and heart failure) at 2-year.	- A total of 259 patients (9.1%) were classified as MINOCA, and 43.1% were treated with DAPT. - Treatment with DAPT was not associated with a reduction of MACE [HR 1.53, 95%CI (0.78–3.01)].	- Type of MINOCA was not reported. - Type of P2Y_12_ inhibitor and the exact duration of DAPT were not reported. - Analysis from a selected population.
**Plaque erosion**			
EROSION Study (Effective Anti-Thrombotic Therapy Without Stenting: Intravascular Optical Coherence Tomography-Based Management in Plaque Erosion): A 1-Year Follow-Up Report (43)	- Single-center, uncontrolled, prospective study - Patients diagnosed with plaque erosion by OCT and residual diameter stenosis <70% on coronary angiogram were treated with anti-thrombotic therapy without stenting. - The primary endpoint was >50% reduction of thrombus volume at 1-month vs. baseline. Secondary, reduction between 12- vs. 1-month	- Median residual thrombus volume decreased significantly from 1 month to 1 year - 49/53 (92.5%) patients remained free from MACE for ≤ 1 year, 3 (5.7%) patients required revascularization because of exertional angina	- Ticagrelor was the only P2Y_12_ inhibitor used - Surrogate primary outcomes (thrombus volume)
Predictors of non-stenting strategy for acute coronary syndrome caused by plaque erosion: 4-year outcomes of the EROSION study (44)	- Long-term follow-up of the EROSION study. -Patients were divided into two groups: TLR and the non-TLR group. - Determine predictors of non-stenting	−11 patients underwent TLR - Patients with better response to antithrombotic therapy in the first month were less likely to require stent implantation during the next 4 years.	- Secondary analysis with a small sample size - Not powered for clinical endpoints
**SCAD**			
Antiplatelet therapy in patients with conservatively managed spontaneous coronary artery dissection from the multicentre DISCO registry (46)	- Observational, international, multicenter, retrospective registry that enrolled patients with SCAD - Patients were classified as receiving SAPT or DAPT. - Primary endpoint was MACE at 12 -month	−67 (33.7%) were given SAPT and 132 (66.3%) with DAPT.- DAPT was associated with a higher risk of MACE compared to SAPT [18.9 vs. 6.0%, HR 2.62; 95%CI (1.22–5.61)].	- Retrospective registry of selected centers. - Prasugrel was used only in one patient (0.7%).
**Epicardial coronary spasm**			
Impact of low-dose aspirin on coronary artery spasm as assessed by intracoronary acetylcholine provocation test in Korean patients (55)	- Patients who undergone acetylcholine provocative test were classified according to the intake of low dose aspirin or not. - Determine the predictors of coronary artery spasm.	- Low-dose aspirin was more frequently associated with CAS and ischemic symptoms, as well as severe and multivessel spasm [OR 1.6, 95%CI (1.0–2.3)].	- Significant baseline differences may also explain the higher rate of spasm in the low-dose aspirin group.
Clopidogrel plus Aspirin Use is Associated with Worse Long-Term Outcomes, but Aspirin Use Alone is Safe in Patients with Vasospastic Angina: Results from the VA-Korea Registry, A Prospective Multi-Center Cohort (48)	- Observational prospective registry of patients with chest pain suggestive of vasospastic angina who received angiography and an ergonovine provocation test. - Patients were classified according to the treatment with aspirin, clopidogrel, both, or none. - Primary endpoint was the composite of all-cause death, ACS, and new-onset symptomatic arrhythmia at 3-year follow-up.	- Patients treated with aspirin and clopidogrel were at a higher risk of adverse events than those treated with non-antiplatelet [10.8 vs. 4.4%; HR 2.41, 95%CI (1.32–4.40)]. - Aspirin-alone group had a similar risk of adverse events than the no-antiplatelet agent group [HR 0.96, 95%CI (0.59–1.55)].	- Clopidogrel was the only P2Y_12_ inhibitor used. - Remaining confounders may have an impact on the estimated risk and outcomes.
Impact of aspirin use on clinical outcomes in patients with vasospastic angina: a systematic review and meta-analysis (47)	- Systematic review and meta-analysis of observational studies up to October of 2020. - Patients with VSA treated with aspirin vs. no aspirin or placebo. - Primary endpoint was MACE.	−6 studies including 3,661 patients (aspirin group, *n* = 1,695; no aspirin group, *n*=, 1966) were analyzed. - Aspirin use may not reduce the risk of future cardiovascular events in VSA patients without significant stenosis [OR 0.90, 95%CI (0.55–1.68), *I*^2^ = 82.2%].	- No randomized clinical trials were included. - Majority of patients presented with angina without MI. - A high heterogenicity among studies was found.
**Takotsubo syndrome (TTS)**			
Prognostic impact of antiplatelet therapy in Takotsubo syndrome: a systematic review and meta-analysis of the literature (50)	- Systematic review and meta-analysis of observational studies up to August of 2020. - Patients with TTS treated with aspirin or P2Y_12_ inhibitors (eventually as DAPT) vs. no treatment. - Primary endpoint was MACE or MACCE at 12-month follow-up.	−6 studies including 1,997 patients were analyzed. - Antiplatelet therapy led to a significantly higher incidence of the composite outcome [OR 1.54; 95%CI (1.09–2.17); *I*^2^ = 0%] and overall mortality [OR 1.72; 95%CI (1.07–2.77); *I*^2^ = 0%].	- No randomized clinical trials were included. - A low heterogenicity among studies was found.
Antiplatelet therapy in Takotsubo cardiomyopathy: does it improve cardiovascular outcomes during index event? (49)	- Single-center retrospective registry of TTS patients. -In-hospital medication was evaluated, including aspirin, clopidogrel or both - Primary endpoint was in-hospital MACE.	−206 patients were included. - Aspirin [OR 0.4, 95%CI (0.16–0.9)] and DAPT [OR 0.23; 95%CI (0.1–0.55)] at the time of hospitalization were independent predictors of a lower rate of MACE.	- Remaining confounders may have an impact on the estimated risk and outcomes. - Only in-hospital outcomes were reported.

*MINOCA, myocardial infarction with non-obstructive coronary arteries; SWEDEHEART, Swedish Web-system for Enhancement and Development of Evidence-based care in Heart disease Evaluated According to Recommended Therapy; MI, myocardial infarction; DAPT, dual antiplatelet therapy; HR, hazard ratio; CI, confidence interval; MACE, major adverse cardiac event; CURRENT-OASIS 7, clopidogrel and aspirin optimal dose usage to reduce recurrent events–seventh organization to assess strategies in ischaemic symptoms; CV, cardiovascular; CAD, coronary artery disease; ACS, acute coronary syndrome; STEMI, ST-segment elevation myocardial infarction; PCI, percutaneous cardiac intervention; PURSUIT, Receptor Suppression Using Integrilin Therapy trial; NSTEMI, non-ST-segment elevation myocardial infarction; OCT, optical coherence tomography; TLR; target lesion revascularization; EROSION, new in vivo diagnosis and paradigm shift in the treatment of patients with acute coronary syndrome study; SCAD, spontaneous coronary artery dissection; SAPT, single antiplatelet therapy; OR, odds ratio; CAS, coronary artery spasm; VSA, vasospastic angina; TTS, Takotsubo syndrome*.

Two secondary analyses of RCTs have assessed the effect of antiplatelet therapy in MINOCA (Table 2). In the Clopidogrel and Aspirin Optimal Dose Usage to Reduce Recurrent Events–Seventh Organization to Assess Strategies in Ischaemic Symptoms (CURRENT-OASIS 7) trial, a total of 23,783 patients with MI were included, and 1,599 (6.7%) had MINOCA. In the CURRENT-OASIS 7 trial, compared with a standard clopidogrel-based DAPT regimen, an intensified dosing strategy appears to offer no additional benefit with a signal of possible harm. In MINOCA patients, no difference in bleeding events was found (35). Moreover, in the Receptor Suppression Using Integrilin Therapy (PURSUIT) trial, a total of 5,767 patients with NSTEMI were enrolled, and 366 (6%) had MINOCA. Patients with MINOCA did not benefit from treatment with eptifibatide, while patients with obstructive CAD did have a benefit. In MINOCA patients, no increase in bleeding events was found (36).

Several retrospective studies have assessed the effect of secondary prevention on clinical outcomes in patients with MINOCA (37–39). In none of these studies, the treatment with DAPT has been associated with a decrease in major adverse cardiovascular events (MACE).

**Figure 5:**
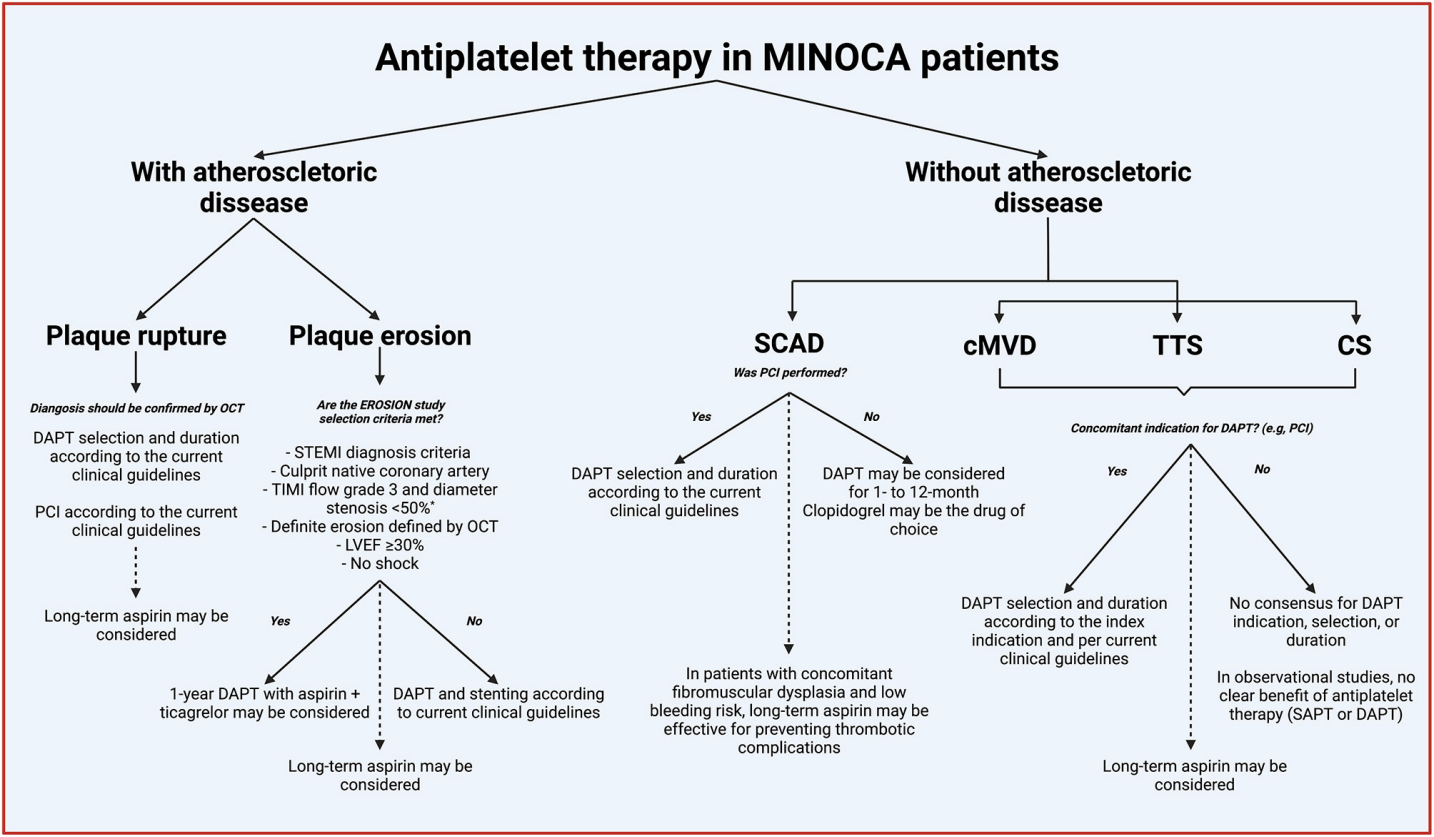
Indications of antiplatelet therapy in patients with MINOCA. *Continued lines* denoted acute or short-term treatment, and *dashed lines* indicate long-term (beyond 1-year). *% of coronary diameter of stenosis adapted to match the MINOCA diagnosis criteria. MINOCA, myocardial infarction with non-obstructive coronary arteries; OCT, optical coherence tomography; DAPT, dual antiplatelet therapy; STEMI, ST-segment elevation myocardial infarction; TIMI, Thrombolysis In Myocardial Infarction; EROSION, new *in vivo* diagnosis and paradigm shift in the treatment of patients with acute coronary syndrome study; LVEF, left ventricular ejection fraction; SCAD, spontaneous coronary artery dissection; PCI, percutaneous cardiac intervention; cMVD, coronary microvascular disease; TTS, Takotsubo syndrome; CS, epicardial coronary spasm; SAPT, single antiplatelet therapy.

Overall, the current evidence on the role of antiplatelet in MINOCA patients is derived from registries or secondary analysis of RTCs, and thus of low quality (5, 35–41). However, the available evidence suggests that antiplatelet therapy, mainly DAPT, is not associated with improvement in clinical outcomes. In the Figure 5, we summarize the potential indications of antiplatelet therapy in patients with MINOCA based on the available scientific evidence.

### Coronary Atherosclerotic Causes of MINOCA

#### Plaque Erosion

The New *in Vivo* Diagnosis and Paradigm Shift in the Treatment of Patients With Acute Coronary Syndrome (EROSION) study was a pilot study that assessed the role of DAPT without stenting in patients with an MI secondary to plaque erosion documented by means of optical coherence tomography (OCT) (42). At 30-day follow-up, DAPT with aspirin and ticagrelor was associated with a significant reduction in thrombus volume and a low rate of adverse events. Furthermore, at 1-year follow-up, 92.5% of patients with MI caused by plaque erosion managed with DAPT without stenting remained free of major adverse cardiovascular events. One patient had to discontinue DAPT because of gastrointestinal bleeding (43). In the final 4-year report, 21% of the patients underwent target lesion revascularization (TLR) (44). The EROSION study provides compelling data supporting the efficacy of DAPT with aspirin and ticagrelor in eroded plaques. However, this was a pilot non-randomized and open-label study with a surrogate primary endpoint. Therefore, this hypothesis should be properly confirmed in dedicated RCTs powered for clinical outcomes.

### Coronary Non-atherosclerotic Causes of MINOCA

#### Spontaneous Coronary Artery Dissection

The role of DAPT in patients with SCAD is a topic of debate. Some experts advocate that DAPT may increase the risk of bleeding and propagation of the hematoma/dissection plane, whereas others claim that the intimal tear can be prothrombotic and the adjunctive use of clopidogrel on top of aspirin may be justified (45). Data from the “DIssezioni Spontanee COronariche” (DISCO) registry suggests that in patients managed conservatively, DAPT may be associated with worse clinical outcomes compared to single antiplatelet therapy (SAPT) (46). The authors evaluated MACE, defined as all-cause death, non-fatal MI, and any unplanned percutaneous coronary intervention (PCI), at 12 months. DAPT, mainly with aspirin and clopidogrel (63%), was associated with an increase in MACE compared to SAPT with aspirin (93%). This difference was driven by re-infarction and urgent revascularization occurring early after the initial SCAD presentation (Table 2). Currently, there are no randomized clinical trials on this topic, and the available evidence suffers from the limitations inherent to retrospective registries.

#### Epicardial Coronary Artery Spasm

Although vasospastic angina has been a topic of continuous research, specific data of MINOCA secondary to coronary vasospasm are limited. Lin et al. reported a systematic review and meta-analysis assessing the role of low-dose aspirin in patients with vasospastic angina without significant CAD (47). The authors included six studies evaluating the outcomes in 3,661 patients. The primary endpoint was MACE, defined as cardiac death, acute coronary syndrome (ACS) and hospitalization due to unstable angina, PCI, symptomatic arrhythmia, appropriate implantable cardioverter-defibrillator, and shock. Aspirin was not associated with a reduction in MACE. However, the quality of the included studies was low, as none was a randomized clinical trial. Moreover, there was very high heterogeneity in the treatment effect within the included studies.

The role of DAPT in coronary vasospasm was evaluated prospectively in the multicenter VA-Korea registry (48). The investigators compared the effect of DAPT with aspirin and clopidogrel vs. aspirin alone on MACE. The primary endpoint was time to composite events of all-cause death, ACS, and symptomatic arrhythmia at 3-year follow-up. Patients treated with DAPT had worse clinical outcomes compared to those treated with aspirin (Table 2). Patients who presented with ACS and smokers had a higher risk of cardiovascular events. However, because this is non-randomized, the possibility of remaining confounding by indication cannot be ruled out.

#### Takotsubo Syndrome

To date, there are mixed findings with regards to the role of antiplatelet therapy in TTS. In a single-center retrospective registry of TTS patients evaluating the in-hospital clinical outcomes, the authors found that DAPT with aspirin and clopidogrel was associated with a lower incidence of MACE during hospitalization (Table 2) (49). MACE was defined as in-hospital heart failure, in-hospital death, stroke, or respiratory failure requiring mechanical ventilation. Nevertheless, the small sample size and the retrospective methodology are significant limitations to be considered.

On the other hand, in a systematic review and meta-analysis including almost two thousand patients, DAPT was associated with an increase in cardiovascular events and mortality (50). Bleeding rates were not reported. However, the quality of the included studies is low, as no randomized trials were included.

### Gaps in the Evidence

The role of antiplatelet therapy in MINOCA continues to be poorly understood. Currently, most of the scientific evidence and guidelines recommendations are supported by low-quality studies. Of note, there is no RCT assessing the role of antiplatelet therapy in the whole cohort of MINOCA patients nor any of its specific etiologies. In clinical practice, most of the MINOCA management is based and extrapolated from studies of patients with obstructive CAD. RCT in the domain of MINOCA is eagerly needed to determine the role of antiplatelet therapy.

In Table 3, we identified current gaps in evidence and proposed potential research strategies that may help provide meaningful data on the role of antiplatelet therapy in MINOCA patients. First, mechanistic studies evaluating platelet function in each type of MINOCA etiology may provide a better understanding of the relationship of the platelet with the disease and identify potential therapeutic targets. Second, prospective clinical studies reporting the use of antiplatelet therapy (type, timing, and duration) and its association with clinical outcomes according to the different etiologies could help assess the effect of antiplatelet therapy in MINOCA patients. Third, dedicated RCTs are crucially needed for evaluating the safety and efficacy of oral and parenteral antiplatelet agents across the different etiologies.

**Table 3:** Current gaps in the evidence and potential research opportunities in the MINOCA field.

	**Gap in the evidence**	**Potential research opportunity**
**MINOCA**		
Pathophysiology	- There is no clear relationship of platelet function with some etiologies such as coronary spasm, TTS, SCAD. - Pathophysiological mechanism of platelet in some etiologies are not completely understood (e.g., coronary microcirculation dysfunction).	- Mechanistic analysis of platelet function according to the different etiologies. - Prospective clinical studies reporting the use of antiplatelet therapy (type, timing, and duration) and its association with clinical outcomes according to the different etiologies.
Treatment	- Evidence suggesting the null effect of antiplatelet therapy comes from registries including a mix of etiologies. - Parenteral antiplatelet therapy has been scarcely tested.	- Dedicated RCTs assessing the effect of antiplatelet therapy (type, timing, and duration) across the different etiologies. - Pilot trial assessing the safety and efficacy of parenteral antiplatelet agents in the acute phase.
**Plaque disruption**		
Treatment of plaque disruption	- Role of invasive (revascularization) vs. medical treatment (DAPT + OMT) is not clear.	- Dedicated RCT comparing an invasive treatment (revascularization) vs. antiplatelet therapy (DAPT) on top of OMT in patients in which plaque rupture or erosion is diagnosed by intracoronary imaging.
**Coronary microcirculation dysfunction**		
Pathophysiology	- Mechanism and pathways relating to platelet function and microcirculation dysfunction is not entirely known.	- Mechanistic analysis of platelet function in patients who develop microvascular dysfunction secondary to reperfusion injury or endothelial dysfunction.
Treatment	- Role of antiplatelet therapy in the prevention and treatment of microcirculation dysfunction is unclear.	- Dedicated RCT comparing antiplatelet therapy (iv or oral) vs. standard of care treatment for preventing and treating microvascular dysfunction in risk of developing it.
**SCAD**		
Treatment	- Role of DAPT vs. SAPT is not clear. - Optimal type, timing, and duration of DAPT or SAPT is not completely understood. - Role of potent P2Y_12_ inhibitors in DAPT or SAPT is poorly understood.	- Dedicated RCT comparing SAPT vs. DAPT in patients with SCAD treated conservatively. Pre-specified analysis of optimal type, timing, and duration of antiplatelet regimens.
**Coronary embolism or** ***in-situ*** **thrombosis**		
Epidemiology	- Incidence of embolism and thrombosis according to different etiologies is unclear.	- Prospective registries evaluating the incidence of embolism and thrombosis according to different etiologies and their relationship with hypercoagulative states.
Pathophysiology	- Mechanism and pathways of microthombosis (without epicardial involvement) is still not completely understood.	- Mechanistic analysis of platelet function in patients who presented microthombosis secondary to embolism or thrombosis.
Treatment	- Role of different antiplatelet agent regimens for the prevention and treatment is unclear.	- Dedicated RTC comparing current standard of care vs. combination of antiplatelet and anticoagulant therapy.
**Epicardial coronary spasm**		
Pathophysiology	- Mechanism and pathways relating to platelet function and coronary spasm is unclear. - Most of the data comes from vasospastic angina, and few are available in patients who develop MI. - Role of antiplatelet therapy SAPT (aspirin) or DAPT during the acute event and long term is unknown.	- Mechanistic analysis of platelet function in patients who develop MI after a coronary spasm. - Dedicated RCT comparing no antiplatelet therapy vs. aspirin vs. DAPT in patients who develop MI after a coronary spasm.
**Takotsubo syndrome**		
Pathophysiology	- Mechanism and pathways relating to platelet function in the etiology of TSS is still a matter of debate.	- Mechanistic analysis of platelet function in TTS.
Treatment	- Role of the antiplatelet agents in TTS is not completely understood.	- Dedicated RCT comparing no antiplatelet therapy vs. aspirin vs. DAPT in TTS patients.

*MINOCA, myocardial infarction with non-obstructive coronary arteries; TTS, Takotsubo syndrome; SCAD, spontaneous coronary artery dissection; RCT, randomized controlled trial; OMT, optimal medical therapy; SAPT, single antiplatelet therapy; MI, myocardial infarction*.

## Future Perspective

In the past years, the scientific community has dedicated efforts to increasing the awareness and refining the diagnosis workflow of MINOCA. However, in the coming years, research will be dedicated to identifying the underlying mechanism and determining the safety and efficacy of potential therapies. Indeed, a shift of current research efforts and priorities from the diagnosis to the treatment area is necessary for improving the prognosis in patients with MINOCA. Table 4 summarizes the ongoing RCTs in which antiplatelet agents are tested in patients with MINOCA. One of the most interesting trials is the Randomized Study of Beta-Blockers and Antiplatelets in Patients With Spontaneous Coronary Artery Dissection (BA-SCAD) trial (51). The investigator will randomize 600 patients in a 2 × 2 factorial design evaluating the safety and safety of beta-blockers and DAPT in patients with SCAD. The compared DAPT regimens consist of 1- vs. 12- month strategies initiated on the day of randomization. Of note, only patients conservatively managed are allowed to be randomized in the DAPT stratum. In the short antiplatelet arm, aspirin monotherapy for 1-month is the recommended regimen. However, the use of DAPT for a period no longer than 1-month is allowed. In the prolonged arm, DAPT with aspirin and clopidogrel for 1-year, followed by aspirin alone, is recommended. Even though ticagrelor and prasugrel are preferred over clopidogrel in ACS patients according to practice guidelines, there are no data for their use in SCAD (3, 51), Nevertheless, in the BA-SCAD trial, the choice of P2Y_12_ inhibitor is at the discretion of the treating physician in line with current clinical guidelines (3).

**Table 4:** Ongoing clinical trials assessing the role of antiplatelet therapy in MINOCA.

	**Objective**	**Methodology**	**Comments**
**MINOCA**			
Etiologic Mechanisms, Myocardial Changes and Prognosis of Patients With MINOCA (NCT04538924) (56)	- To assess the etiologic mechanisms of myocardial damage in patients with MINOCA and evaluate various therapeutic strategies for these patients.	- Parallel assignment RCT (1:1) - Patients will be assigned to traditional MI treatment (statin, ACEI/ARB), beta-blockers, and DAPT) or to the experimental arm of low-dose statin and ACEI/ARB. In the case of vasospasm, calcium channel blockers. -Sample size 150 patients. - Primary endpoint is all-cause death at 1-year.	- DAPT will be tested among the conventional treatment, but not individually. - Pilot study, small sample size.
**SCAD**			
Randomized Study of Beta-Blockers and Antiplatelets in Patients With Spontaneous Coronary Artery Dissection (BA-SCAD) (NCT04850417) (51).	- To assess the efficacy of pharmacological therapy (beta-blockers and antiplatelet) in patients with SCAD.	- Factorial 2 × 2 RCT (1:1/1:1) - Patients will be allocated to beta-blockers (yes/no) and b)“short” (1 month) vs. “prolonged” (12 months) antiplatelet therapy. - Sample size 600 patients. - Primary endpoint includes a composite of death, myocardial infarction, stroke, coronary revascularization, recurrent SCAD, and unplanned hospitalization for acute coronary syndrome or heart failure at 1 year.	- Large sample size trial that will assess the effect of short vs. standard DAPT (aspirin and clopidogrel). - Patients with reduced ejection fraction can be randomized in the beta-blocker stratum and only patients conservatively managed patients will be randomized to the antiplatelet stratum.
**Takotsubo syndrome**			
BROKEN-SWEDEHEART-Optimized Pharmacological Treatment for Broken Heart (Takotsubo) Syndrome (NCT04666454).	- To document an optimized pharmacologic treatment (adenosine, dipyridamole, and apixaban) for patients with Takotsubo Syndrome.	- Factorial 2 × 2 randomized registry clinical trial (1:1/1:1). - Patients can be assigned to - Randomization 1 active: Adenosine + dipyridamole - Randomization 1 control: Treatment as per ESC - TTS guidelines - Randomization 2 active: Apixaban 5 mg c/12 h - Randomization 2 control: No intervention - Sample size 1,000 - Primary endpoints: - Randomization 1: First co-primary endpoint: Wall -motion score index at 72 h - Randomization 1: Second co-primary endpoint: -Composite of death, cardiac arrest, or the need for -cardiac mechanical assist device, or re- hospitalization for heart failure or ejection fraction <50% at 30 days - Randomization 2: occurrence of any thromboembolic event or death, or the presence of a cardiac thrombus, as assessed by echocardiography at 30 days.	- Large sample size trial. - RCT nested on a registry. - Dipyridamole is mainly used for its effect on the adenosine recapture rather than its antiplatelet effect.

*MINOCA, myocardial infarction with non-obstructive coronary arteries; RCT, randomized controlled trial; ACE, angiotensin-converting enzyme; ARBs, Angiotensin II receptor blockers; DAPT, dual antiplatelet therapy; SCAD, spontaneous coronary artery dissection; ESC, European society of cardiology; TTS, Takotsubo syndrome*.

## Conclusions

The role of platelets in the pathophysiology of MINOCA is not entirely understood. Although in some of its causes such as plaque disruption, the role of platelets in the pathophysiological mechanism is well-described, in other etiologies such as coronary artery vasospasm or TTS, this relationship remains unclear. Nevertheless, the description of these mechanisms is critical for identifying potential therapeutic targets. The current low-quality evidence suggests that antiplatelet therapy may not be associated with better clinical outcomes in MINOCA patients. Especially in non-atherosclerotic causes, the utility of antiplatelet agents is questionable. However, in the absence of dedicated RCTs, the role of antiplatelet therapy in MINOCA is a matter of debate, and the current recommendations for antiplatelet therapy are based on registries and expert opinion. The identification of underlying mechanisms linking the different MINOCA etiologies with platelet function is necessary. Ultimately, dedicated RCTs to assess the safety and efficacy of antiplatelet agents in MINOCA patients and specific etiologies are warranted.

## Author Contributions

LO-P and DJA contributed to conception and design of the review and wrote the first draft of the manuscript. MG, DC, and SB wrote sections of the manuscript. All authors contributed to manuscript revision, read, and approved the submitted version.

## Conflict of Interest

DC declares that he has received consulting and speaker's fee from Amgen, Arena, Biotronik, Daiichi Sankyo, Sanofi, Terumo, outside the present work. SB declares that he is consultant from Boston Scientific and iVascular and he received speaker's fee from Abbott Vascular, outside the present work. DJA declares that he has received consulting fees or honoraria from Abbott, Amgen, Aralez, AstraZeneca, Bayer, Biosensors, Boehringer Ingelheim, Bristol-Myers Squibb, Chiesi, Daiichi-Sankyo, Eli Lilly, Haemonetics, Janssen, Merck, PhaseBio, PLx Pharma, Pfizer, Sanofi, and The Medicines Company and has received payments for participation in review activities from CeloNova and St Jude Medical. DJA also declares that his institution has received research grants from Amgen, AstraZeneca, Bayer, Biosensors, CeloNova, CSL Behring, Daiichi-Sankyo, Eisai, Eli Lilly, Gilead, Idorsia, Janssen, Matsutani Chemical Industry Co., Merck, Novartis, Osprey Medical, Renal Guard Solutions, and the Scott R. MacKenzie Foundation. The remaining authors declare that the research was conducted in the absence of any commercial or financial relationships that could be construed as a potential conflict of interest.

## Publisher's Note

All claims expressed in this article are solely those of the authors and do not necessarily represent those of their affiliated organizations, or those of the publisher, the editors and the reviewers. Any product that may be evaluated in this article, or claim that may be made by its manufacturer, is not guaranteed or endorsed by the publisher.

## Abbreviations

ACS: acute coronary syndrome
ADP: adenosine diphosphate
AHA: American Heart Association
CAD: coronary artery disease
CURRENT-OASIS 7: clopidogrel and aspirin optimal dose usage to reduce recurrent events–seventh organization to assess strategies in ischaemic symptoms
DAPT: dual antiplatelet therapy
EROSION: new *in vivo* diagnosis and paradigm shift in the treatment of patients with acute coronary syndrome study
ESC: European Society of Cardiology
GP: glycoprotein
IIb/IIIa: αIIβ3 integrin
IMR: index of microcirculatory resistance
MACE: major adverse cardiovascular events
MI: myocardial infarction
MINOCA: myocardial infarction with non-obstructive coronary arteries
NETs: neutrophil extracellular traps
NSTEMI: non-ST-segment elevation myocardial infarction
OCT: optical coherence tomography
PCI: percutaneous coronary intervention
PURSUIT: receptor suppression using integrilin therapy trial
RCTs: randomized controlled trials
SAPT: single antiplatelet therapy
SCAD: spontaneous coronary artery dissection
STEMI: ST-segment elevation myocardial infarction
TIMI: thrombolysis in myocardial infarction
TTS: takotsubo syndrome
TXA_2_: thromboxane A_2_
vWF: von Willebrand factor.
